# Practical Implications of the Update to the 2015 Japan Standard Population: Mortality Archive From 1950 to 2020 in Japan

**DOI:** 10.2188/jea.JE20220302

**Published:** 2023-07-05

**Authors:** Hirokazu Tanaka, Sayo Tanaka, Kayo Togawa, Kota Katanoda

**Affiliations:** Division of Surveillance and Policy Evaluation, Institute for Cancer Control, National Cancer Center, Tokyo, Japan

**Keywords:** standard population, age-standardized mortality, the 2015 Japan Standard Population, the 1985 Japan Standard Population, direct standardization method

## Abstract

**Background:**

The 2015 Japan Standard Population (JSP) was established in response to changes in the age structure. However, the effects of major updates, especially the recategorization of older age groups, for interpreting various health metrics have not been clarified.

**Methods:**

Population data were collected and estimated for older age categories (85–89, 90–94, and ≥95 years). Data on the number of deaths were also collected from the Vital Statistics. We recalculated the all-cause and leading cause-specific age-standardized mortality rate (ASMR) using the 2015 JSP by the direct standardization method for data from 1950 to 2020. We compared ASMRs calculated using the 2015 JSP with those calculated using the 1985 JSP. Pearson’s correlation coefficients were used to evaluate the consistency of mortality trends between the 2015 and 1985 JSPs.

**Results:**

The absolute all-cause ASMRs calculated using the 2015 JSP were 2.22–3.00 times higher than those calculated using the 1985 JSP. The ASMR ratios increased gradually over time. While trends in all-cause and cause-specific ASMRs calculated using the 2015 JSP and 1985 JSP were generally highly correlated (Pearson’s correlation coefficient [*r*] = 0.993 for all-cause), correlations were relatively low for malignant neoplasms (*r* = 0.720 for men and *r* = 0.581 for women) and pneumonia/bronchitis (*r* = 0.543 for men and *r* = 0.559 for women) due to non-monotonous trends over time and fluctuations in earlier time periods.

**Conclusion:**

The effect of introducing the new JSP for interpreting trends in all-cause mortality was considered minimal. However, caution is needed when interpreting trends in some cause-specific mortality rates.

## INTRODUCTION

Age standardization is a typical method used to compare health metrics, such as disease incidence, prevalence, and mortality rate, across different time points or age structures.^1^ A standard population is the standard by which incidence, prevalence, and mortality rate are calculated with the adjustment of age structure.^2^ To calculate age-standardized mortality rate (ASMR) with direct standardization method, the age-categorized number of deaths and population (commonly 5-year age groups) are required for calculating the crude mortality rate for the age groups (eFigure 1). Then, the crude mortality rate by 5-year age groups would be weighted based on the selected standard population.^1^ For consistency, those in the health sector including public health practitioners should use the same standard population to determine age-standardized incidence and mortality rate.

In Japan, the 1985 Japan Standard Population (JSP) has been the main standard by which Japan’s ASMR and morbidity rate were calculated for the past several decades.^3^^–^^5^ However, in February 2022, the Japanese Ministry of Health, Labour and Welfare (MHLW) released an updated version, the 2015 JSP, in response to changes in the age structure of the population.^6^ The 2015 JSP includes greater weights on older population categories than those of well-known standard populations such as the 1985 JSP, 2013 European Standard Population, 2000 United States Standard Population, World Health Organization (WHO) World Standard Population (2000–2025), and Segi’s World Standard Population.^2^^,^^4^^,^^6^^–^^9^ Another major revision is the change in the oldest aggregated age category from “85 years and over” in the 1985 JSP to “95 years and over” in the 2015 JSP.^2^ Because the oldest age category was previously “85 years and over” in official MHLW statistics, this change could affect recalculation of ASMR for past trends.

Annual ASMR data are essential for long-term trend analysis, especially statistical evaluation (eg, estimation of average annual percentage change in ASMR).^10^ However, the MHLW announced that while they published recalculated ASMRs for each year between 2005–2020 in September 2022, they only calculated for every 5 years between 1950–2000 for all-cause death and some specific causes of death only.^6^ Due to the incompleteness of the data, it is still unknown how the change in the standard population affects the long-term trends in all-cause or cause-specific ASMR. Thus, we aimed to (1) develop all-cause and cause-specific annual ASMRs every year from 1950 to 2020 using the best available data for population and number of deaths and (2) investigate the impact of the change in the 2015 JSP from the 1985 JSP.

## METHODS

Population data (“0 years”, “1–4 years” to “80–84 years”) was referenced from the census (every 5 years)^11^ and population estimates (for interval years between the censuses) developed by the Ministry of Internal Affairs and Communications.^12^ We also collected population data for those in older age categories (85–89, 90–94, 95 years and over) using the census (every 5 years).^11^ To estimate the missing population data for older age categories, we used population estimates (for interval years between the censuses) developed by the Ministry of Internal Affairs and Communications^12^ and the National Institute of Population and Social Security Research using the cohort-interpolation method. Numbers of all-cause and cause-specific deaths between 1950–2020 were extracted from the Vital Statistics (Time series vital statistics, Japan; stored on digital versatile disc).^3^ This database contains detailed data on the number of deaths at every age up to 99 years (and an aggregated category of “100 years and over”).^3^ Crude mortality rates were calculated for 5-year age categories (including “0 years”, “1–4 years”, and “95 years and over”) between 1950–2020.

**Figure 1.**
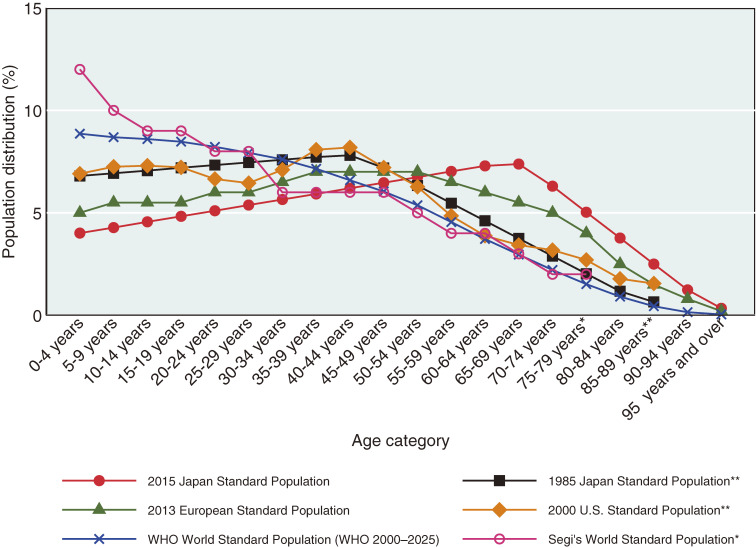
Age distributions of standard populations. ^*^Those 75 years and over are aggregated into one category in the Segi’s World Standard Population. ^**^Those 85 years and over are aggregated into one category in the 1985 Japan Standard Population and the 2000 United States Standard Population

We calculated and compared ASMRs (100,000 per persons) calculated using the 2015 JSP with those calculated using the 1985 JSP, 2013 European Standard Population, 2000 United States Standard Population, WHO World Standard Population (2000–2025), and Segi’s World Standard Population (Table 1 and Figure 1).^2^^,^^4^^,^^6^^–^^9^ ASMRs were calculated based on each standard population using the direct standardization method for the Japanese population.

**Table 1.** The 2015 Japan Standard Population and other standard populations

Age category	2015 Japan Standard Population	1985 Japan Standard Population	2013 European Standard Population	2000 United States Standard Population	WHO World Standard Population (2000–2025)	Segi’s World Standard Population
0 years	978,000	—	1,000	—	—	—
1–4 years	4,048,000	—	4,000	—	—	—
0–4 years	—	8,180,000	—	18,987,000	8,860	12,000
5–9 years	5,369,000	8,338,000	5,500	19,920,000	8,690	10,000
10–14 years	5,711,000	8,497,000	5,500	20,057,000	8,600	9,000
15–19 years	6,053,000	8,655,000	5,500	19,820,000	8,470	9,000
20–24 years	6,396,000	8,814,000	6,000	18,257,000	8,220	8,000
25–29 years	6,738,000	8,972,000	6,000	17,722,000	7,930	8,000
30–34 years	7,081,000	9,130,000	6,500	19,511,000	7,610	6,000
35–39 years	7,423,000	9,289,000	7,000	22,180,000	7,150	6,000
40–44 years	7,766,000	9,400,000	7,000	22,479,000	6,590	6,000
45–49 years	8,108,000	8,651,000	7,000	19,806,000	6,040	6,000
50–54 years	8,451,000	7,616,000	7,000	17,224,000	5,370	5,000
55–59 years	8,793,000	6,581,000	6,500	13,307,000	4,550	4,000
60–64 years	9,135,000	5,546,000	6,000	10,654,000	3,720	4,000
65–69 years	9,246,000	4,511,000	5,500	9,410,000	2,960	3,000
70–74 years	7,892,000	3,476,000	5,000	8,726,000	2,210	2,000
75–79 years (^*^75 and over)	6,306,000	2,441,000	4,000	7,415,000	1,520	2,000^*^
80–84 years	4,720,000	1,406,000	2,500	4,900,000	910	—
85–89 years (^**^85 and over)	3,134,000	784,000^**^	1,500	4,259,000^**^	440	—
90–94 years	1,548,000	—	800	—	150	—
95 years and over (^***^95–99 years)	423,000	—	200	—	40^***^	—
100 years and over	—	—	—	—	5	—
Total	125,319,000	120,287,000	100,000	274,634,000	100,035	100,000

We archived all-cause and leading cause-specific ASMRs (100,000 per persons) calculated using the 2015 JSP from 1950 to 2020. Pearson’s correlation coefficient was used to assess the consistency of new ASMR trends (2015 JSP) with those determined using 1985 JSP. For causes of death with relatively low correlation coefficients, we further analyzed the correlations between the trends of ASMR with the two different JSPs. Causes of death included: tuberculosis, malignant neoplasms, diabetes, hypertension, heart diseases, cerebrovascular diseases, pneumonia and bronchitis, liver disease, senility, accidents, and suicide. These classifications were the same as the leading causes of death included in the official mortality statistics by MHLW.^3^

## RESULTS

**Figure 2.**
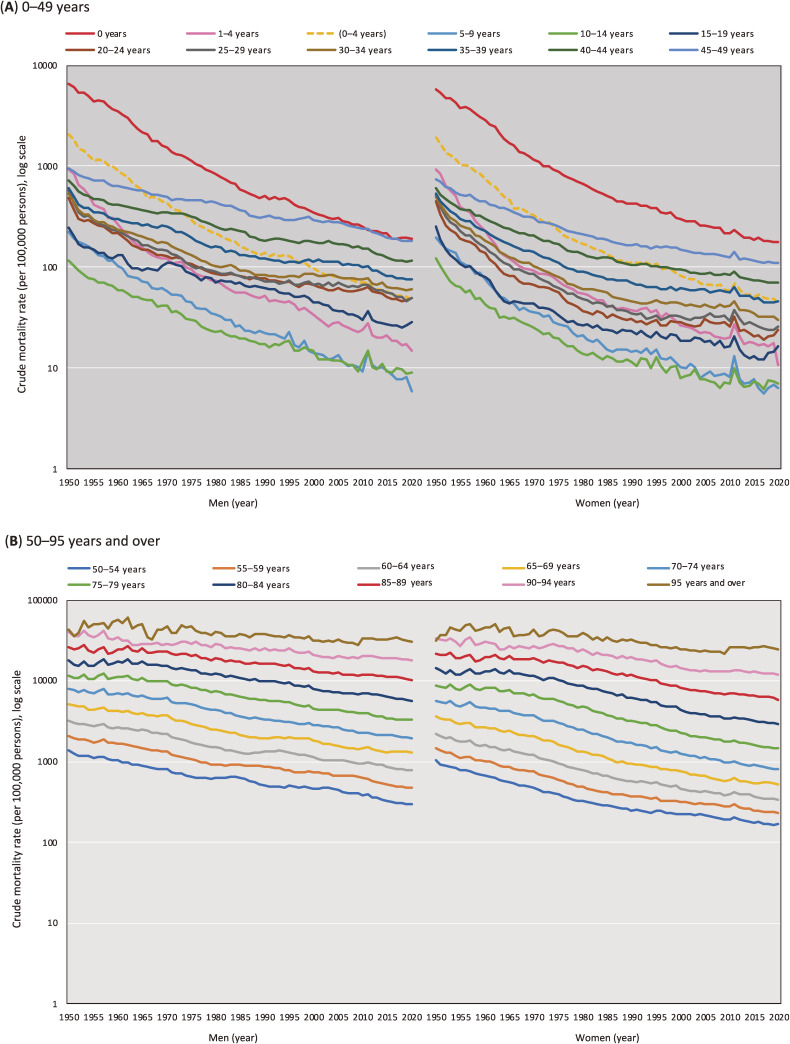
Trends in the crude mortality rate across 5-year age categories (per 100,000 persons, log scale)

Figure 2 shows the trends in the crude mortality rate for the 5-year age categories. We found stable mortality declines across all age categories except for “90–94 years” and “95 year and over,” which were approximately flat for several decades. A mortality spike was observed in 2011, especially among the young population (eg, 5–9 years), when the Great East Japan earthquake occurred.

**Figure 3.**
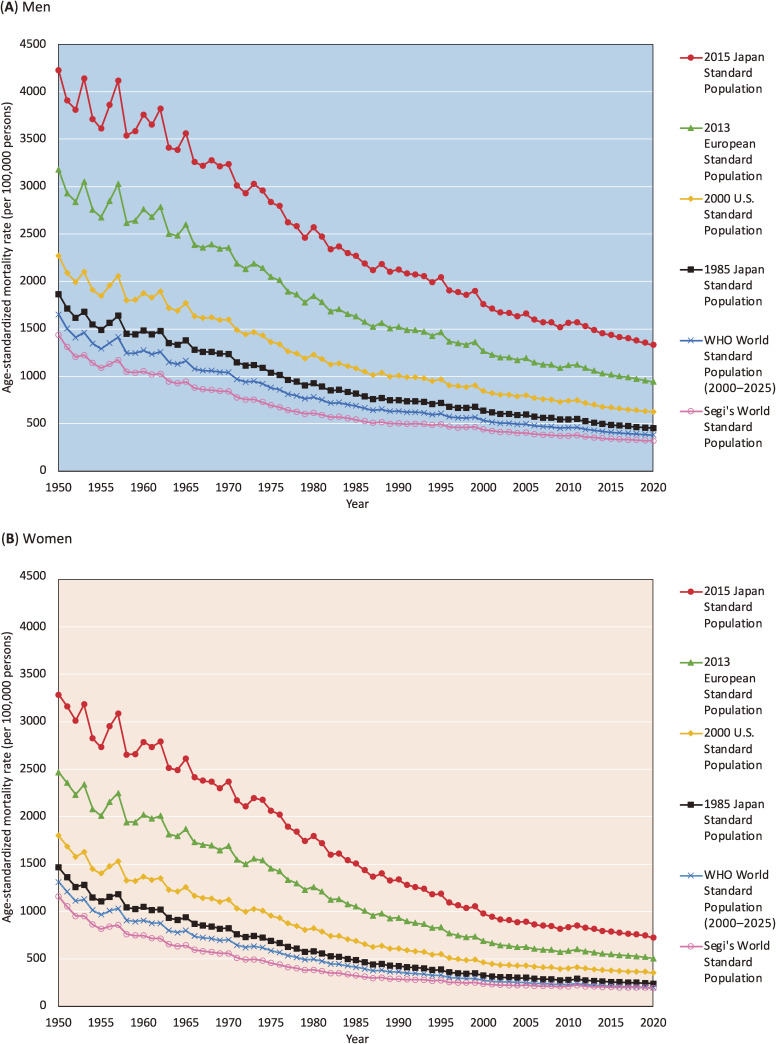
Trends in the all-cause age-standardized mortality rate (ASMR) in Japanese men (**A**) and women (**B**) calculated using six different standard populations (per 100,000 persons)

Figure 3 shows the trends in all-cause ASMR (100,000 per persons) calculated using various standard populations for the Japanese population. ASMRs calculated using the 2015 JSP were higher than those calculated using any other well-known standard population. This finding is reasonable because the 2015 JSP places greater weight on the older age group (high mortality risk group) than any other standard population. All-cause ASMRs calculated with the 2015 JSP were 2.22–3.00 times higher than those calculated with the 1985 JSP. The ASMR ratios increased gradually over time. eTable 1 and eTable 2 provide detailed all-cause and cause-specific ASMRs (100,000 per persons) calculated using the 2015 JSP and the 1985 JSP, respectively.

**Figure 4.**
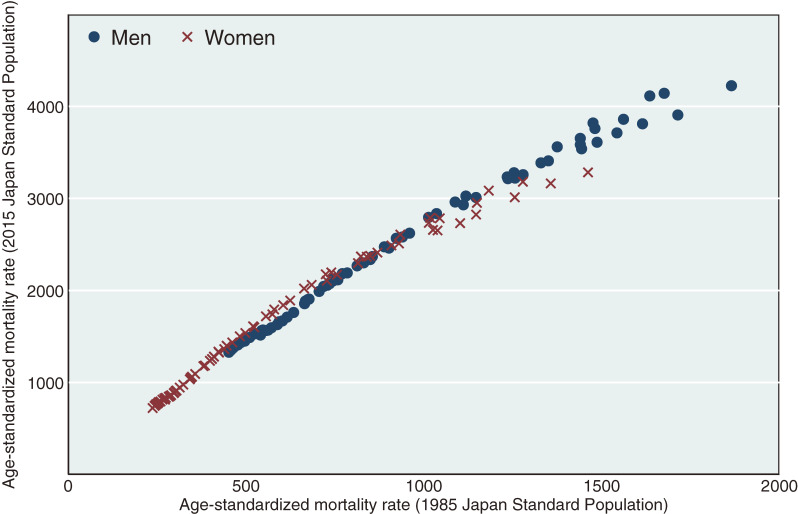
Correlation of all-cause age-standardized mortality rates (per 100,000 persons) calculated using the 1985 Japan Standard Population vs those calculated using the 2015 Japan Standard Population, 1950–2020. Pearson’s correlation coefficients: 0.993 (*P* < 0.01) for both sexes, 0.995 (*P* < 0.01) for men, 0.989 for women (*P* < 0.01)

Figure 4 shows the correlation of all-cause ASMRs calculated using the 1985 JSP compared to the 2015 JSP from 1950 to 2020. ASMRs calculated with the 1985 JSP and 2015 JSP were clearly correlated (Pearson’s correlation coefficients [*r*] = 0.993; *P* < 0.01) for both sexes (*r* = 0.995; *P* < 0.01 for men and *r* = 0.989; *P* < 0.01 for women).

**Figure 5.**
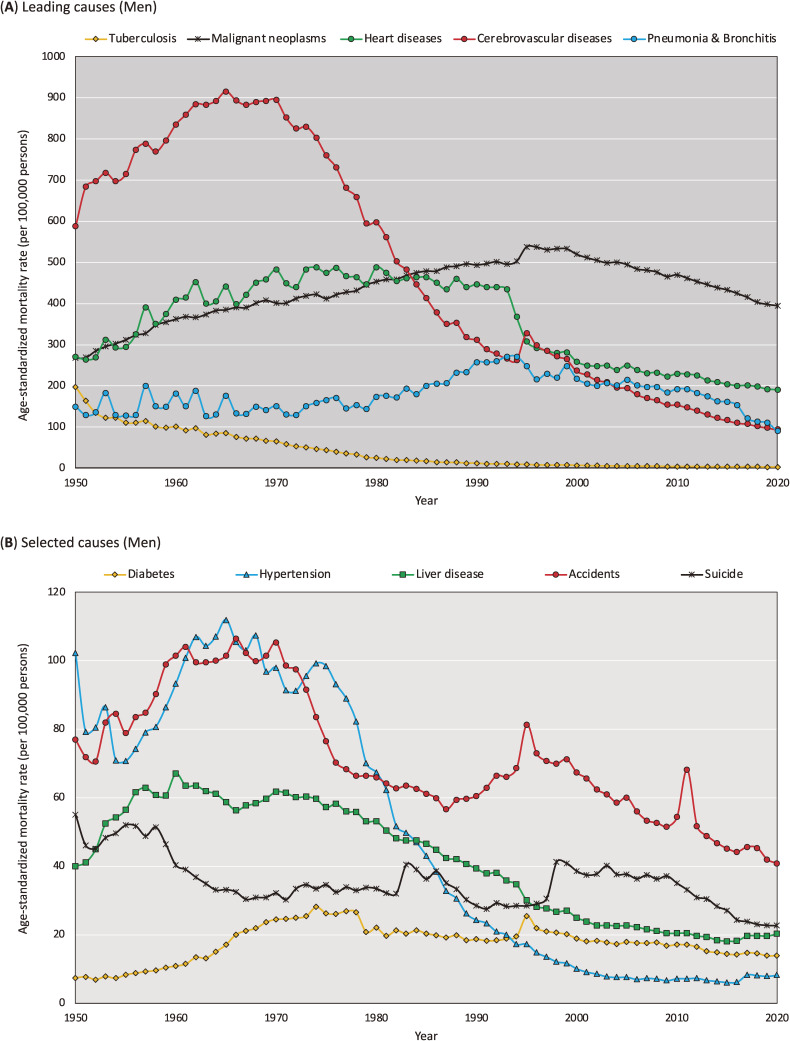
Trends in the cause-specific age-standardized mortality rate in men calculated using the 2015 Japanese Standard population, 1950–2020 (per 100,000 persons)

**Figure 6.**
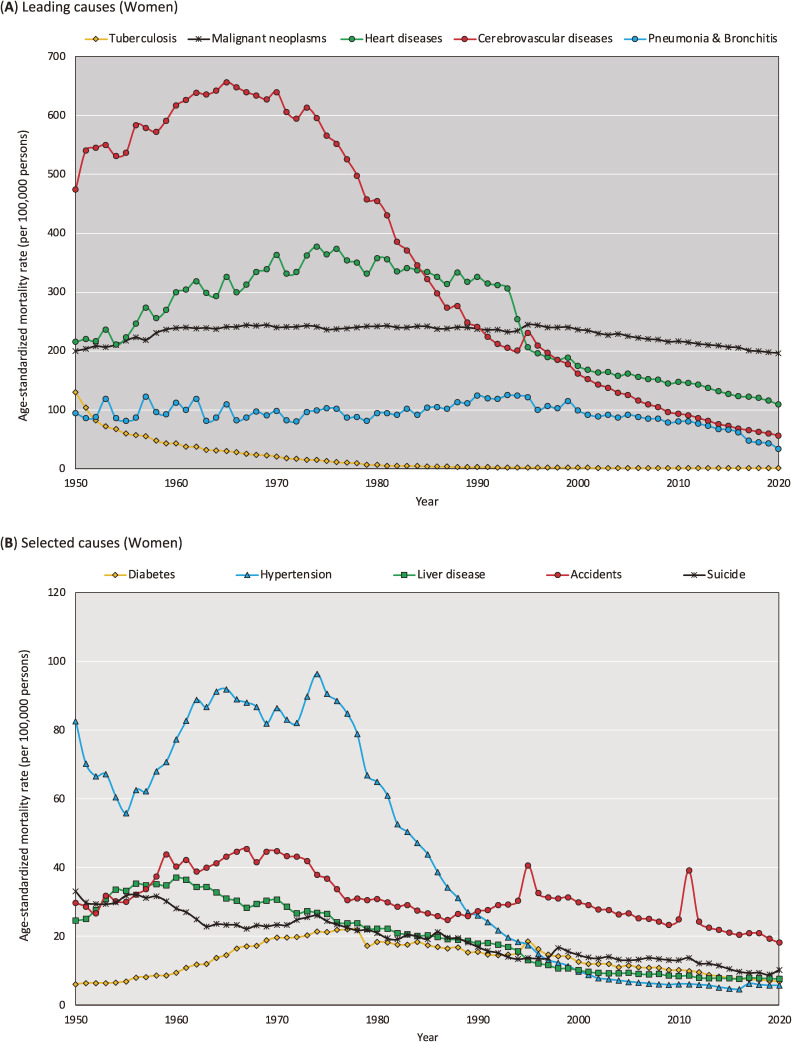
Trends in the cause-specific age-standardized mortality rate in women calculated using the 2015 Japanese Standard population, 1950–2020 (per 100,000 persons)

Figure 5 and Figure 6 show the trends in leading cause-specific ASMRs calculated using the 2015 JSP by sex between 1950 and 2020. Details of the data presented in these figures are shown in eTable 1 and eTable 2.

The results of our additional analysis are presented in eTable 3, which summarizes correlations of cause-specific ASMR between 1985 JSP and 2015 JSP. We found that while Pearson’s correlation coefficients were generally high (eg, cerebrovascular diseases: *r* = 0.991 for men and *r* = 0.983 for women), the results were less consistent for some causes of death, as evidenced by relatively low Pearson’s correlation coefficients for malignant neoplasms (*r* = 0.720 for men and *r* = 0.581 for women) and pneumonia/bronchitis (*r* = 0.543 for men and *r* = 0.559 for women). We further analyzed the correlations of ASMR for malignant neoplasms (eFigure 2) and pneumonia/bronchitis (eFigure 3) to investigate the reasons for the low Pearson’s correlation coefficients. eFigure 2A shows that the ASMR for malignant neoplasms was non-monotonous for both sexes. For men, the increase in ASMR turned into a decrease after 1995, while for women, the slope of the increase changed after 1960. eFigure 2B shows a two-dimensional plot of the relationship, confirming that the correlation between the two trends changed at the above-mentioned inflection points. As shown in eFigure 3A, trends in the ASMR for pneumonia/bronchitis were unstable up to the 1970s. Even in eFigure 3B, there is no clear linear relationship before the year 1979. Thereafter, the linear relationship changed between 1980 and 1992, and after 1993.

## DISCUSSION

Trends in all-cause and cause-specific ASMRs calculated using the 2015 JSP and 1985 JSP were highly correlated (Pearson’s correlation coefficients approximated one), while correlations were relatively low for malignant neoplasms and pneumonia/bronchitis due to non-linear associations caused by fluctuations in mortality trends. While absolute ASMRs calculated using the 2015 JSP were higher than values calculated using any other standard population, trends in all-cause ASMR were similar. We confirmed that all-cause ASMRs calculated using the 2015 JSP were highly correlated with those calculated using the 1985 JSP, indicating that trends in all-cause ASMRs were not distorted by changes made when updating the 1985 JSP to the 2015 JSP. Similar trends in all-cause ASMR were observed when calculated using different standard populations.

While cause-specific ASMRs calculated using the 2015 JSP and 1985 JSP were generally highly correlated, we observed inconsistent trends in cause-specific mortality for malignant neoplasms and pneumonia/bronchitis, indicating the need for careful interpretation of these values. The overall trends in ASMR for malignant neoplasms and pneumonia/bronchitis by sex changed after introducing the 2015 JSP. These changes occurred because the crude mortality rate for malignant neoplasms among 65–69-year-olds (the largest weighted age band in the 2015 JSP) increased in 1950–1995 and decreased in 1995–2020 at a steeper rate than that in 40–44-year-olds (the largest weighted age band in the 1985 JSP).^10^ The inconsistent trends in cause-specific mortality were due to non-monotonous changes in mortality for malignant neoplasms and fluctuations in mortality data for pneumonia/bronchitis.

The new JSP is considered valuable and necessary because it (1) adapts standards to the extremely rapid aging Japanese population, (2) better categorizes older age groups (eg, “85–89 years” and “90–94 years”), and (3) includes a new “0 years” category to account for the high prevalence of health problems among the elderly that have emerged over the past three decades in Japan. For example, about 30% of deaths (113,244/378,385 for both sexes) were due to malignant neoplasms (ICD-10: C00-C97) among the “85 years and older” group in 2020 and 3.3% of deaths were due to malignant neoplasms (12,475/378,385 for both sexes) among the “95 years and older” group in 2020.^3^ While the 1985 JSP included these groups in one aggregated category (“85 years and over”), in the 2015 JSP, they are subdivided (“85–89 years”, “90–94 years”, and “95 years and over”). Thus, the new standard population corresponds better to the hyper-aging society of the Japanese population.

We agree to introduce new standard population in response to rapid changes in the age structure for the Japanese population. However, changing standard populations can raise issues related to comparability which can confuse users when comparing health metrics (eg, mortality rates) produced before and after updates to standard populations. This makes it difficult for users to compare metrics produced before and after updates to standard populations. We propose to the government to prepare the necessary data (eg, detailed population data covering the older age groups for denominator and number of death for numerator) to motivate users to consistently adopt the new JSP for recalculation. In addition, the effects of major updates that were discussed in this study should have been evaluated before the government introduced new standard population. Because mortality statistics are fundamental metrics for public health and the changing standard population have a large impact on health statistics, the frequency of update must be minimized. Indeed, the Surveillance, Epidemiology, and End Results Program managed by National Cancer Institute’s Division of Cancer Control and Population Sciences has clearly stated that they have no plans to change the 2000 United States Standard Population.^13^

The 2015 JSP should be used with caution. Although we were able to calculate ASMR values using the new JSP for national data, similar calculations for prefectural data would be difficult. This is because, in many cases, there are no region-level population data available for the exact age categories used in the 2015 JSP, especially the newly subdivided older age groups (eg, 90–94 years). Although past prefecture-level population data could be updated to cover the older age categories in the future, at this point, continuing to update health metrics using the 1985 JSP is a more realistic approach for comparing prefecture-level mortality or incidence rates.

The strengths of this analysis are the consistency of our findings with official mortality statistics and long-term analysis conducted over 70 years. All-cause ASMRs calculated using the 2015 JSP in this study completely matched the official mortality statistics reported between 2005 and 2020.^3^ It is important to note that the number of deaths and population in older age groups (eg, 90–94 years) fluctuated greatly in the past, especially in 1950–1970. In the 1985 JSP, the effect of these fluctuations was less crucial because older populations were aggregated into a single “85 years and older” group. According to our analysis, ASMRs calculated using the 2015 JSP were more stable after 1970, so they may be more reliable for assessing exact trends in ASMR. Thus, we propose that our mortality archive may be more robust for long-term analysis between 1970 and 2020.

In conclusion, the effect of the introduction of the 2015 JSP on interpreting trends in the all-cause mortality rate was minimal. However, caution is needed when interpreting trends in some cause-specific mortality rates, namely those for malignant neoplasms and pneumonia/bronchitis, which vary depending on the characteristics of past trends.

## SUPPLEMENTARY MATERIAL

eTable 1. Trends in all-cause and cause-specific age-standardized mortality rate calculated using the 2015 Japanese Standard population, 1950–2020 (per 100,000 persons)

eTable 2. Trends in all-cause and cause-specific age-standardized mortality rate calculated using the 1985 Japanese Standard population, 1950–2020 (per 100,000 persons)

eTable 3. Correlation of all-cause and cause-specific age-standardized mortality rates between the 1985 Japan Standard Population and the 2015 Japan Standard Population, 1950–2020; Pearson’s correlation coefficients

eFigure 1. The formula to calculate age-standardized morality rate

eFigure 2. Trends in (**A**) malignant neoplasms age-standardized mortality rates and (**B**) association of age-standardized mortality rates from malignant neoplasms

eFigure 3. Trends in (**A**) pneumonia & bronchitis age-standardized mortality rates and (**B**) association of age-standardized mortality rates from pneumonia & bronchitis

## References

[1] Rothman KJ, Greenland S, Lash TL. *Modern epidemiology*. vol 3. Philadelphia, US: Wolters Kluwer Health/Lippincott Williams & Wilkins; 2008.

[2] The Surveillance, Epidemiology, and End Results (SEER) Program. Standard Populations (Millions) for Age-Adjustment. https://seer.cancer.gov/stdpopulations/; 2020 Accessed 18.10.2022.

[3] The Ministry of Health, Labour and Welfare. Vital Statistics. https://www.mhlw.go.jp/english/database/db-hw/vs01.html; 2022 Accessed 18.10.2022.

[4] The Ministry of Health, Labour and Welfare. Standardised mortality rates. https://www.mhlw.go.jp/toukei/saikin/hw/jinkou/other/00sibou/1.html; 2001 Accessed 18.10.2022.

[5] Dhungel B, Wada K, Tanaka H, Gilmour S. Time to update the Japanese standard population for comparing mortality rates. Arch Public Health. 2022;80(1):153. 10.1186/s13690-022-00908-0 35668536 PMC9169265

[6] The Ministry of Health, Labour and Welfare. Standard Populations for Age-Adjustment. https://www.mhlw.go.jp/toukei/saikin/hw/jinkou/kakutei20/dl/14_nencho.pdf; 2022 Accessed 18.10.2022.

[7] Ahmad OB, Boschi-Pinto C, Lopez AD, Murray CJ, Lozano R, Inoue M. *Age standardization of rates: a new WHO standard*. Geneva: World Health Organization. 2001;9(10):1–14.

[8] Commission E. Revision of the European Standard Population - Report of Eurostat’s task force - 2013 edition. https://ec.europa.eu/eurostat/documents/3859598/5926869/KS-RA-13-028-EN.PDF/e713fa79-1add-44e8-b23d-5e8fa09b3f8f; 2013 Accessed 18.10.2022.

[9] Segi M. *Cancer mortality for selected sites in 24 countries (1950–57)*. Sendai, JP: Department of Public Health, Tohoku University of Medicine; 1960.

[10] Katanoda K, Hori M, Saito E, . Updated trends in cancer in Japan: incidence in 1985–2015 and mortality in 1958–2018—a sign of decrease in cancer incidence. J Epidemiol. 2021;31(7):426–450. 10.2188/jea.JE20200416 33551387 PMC8187612

[11] The Ministry of Internal Affairs and Communications. Population Census. https://www.stat.go.jp/data/kokusei/2015/; 2020 Accessed 18.10.2022.

[12] The Ministry of Internal Affairs and Communications. Population estimation. https://www.stat.go.jp/data/jinsui/index.html; 2020 Accessed 18.10.2022.

[13] The Surveillance, Epidemiology, and End Results (SEER) Program. Use of the 2000 U.S. Standard Population for Age-Adjustment. https://seer.cancer.gov/stdpopulations/2000stdpop-use.html; 2020 Accessed 18.10.2022.

