# Supplementary material for: Practical Implications of the Update to the 2015 Japan Standard Population: Mortality Archive From 1950 to 2020 in Japan
Source: J Epidemiol. 2023 Jul 5;33(7):372–80. doi: 10.2188/jea.JE20220302 (PMC10257988; doi:10.2188/jea.JE20220302)
Supplement: Supplementary file 1 [file je-33-372-s001.zip › JE20220302_eMaterials/@JE20220302_eFigure_acceped_33-5-clean.pdf]

Age-standardized mortality rate (ASMR) is given by

$$ASMR = \sum r_i \left(\frac{n_{i,Standard\ population}}{\sum_i n_{i,Standard\ population}} \right)$$

where r_i refers to the mortality rate for the i_{th} age groups (commonly 5-year age groups) in populations and $n_{i,Standard\ population}$ refers to the number of population in the i_{th} age group of a selected standard population.

eFigure 1. The formula to calculate age-standardized mortality rate

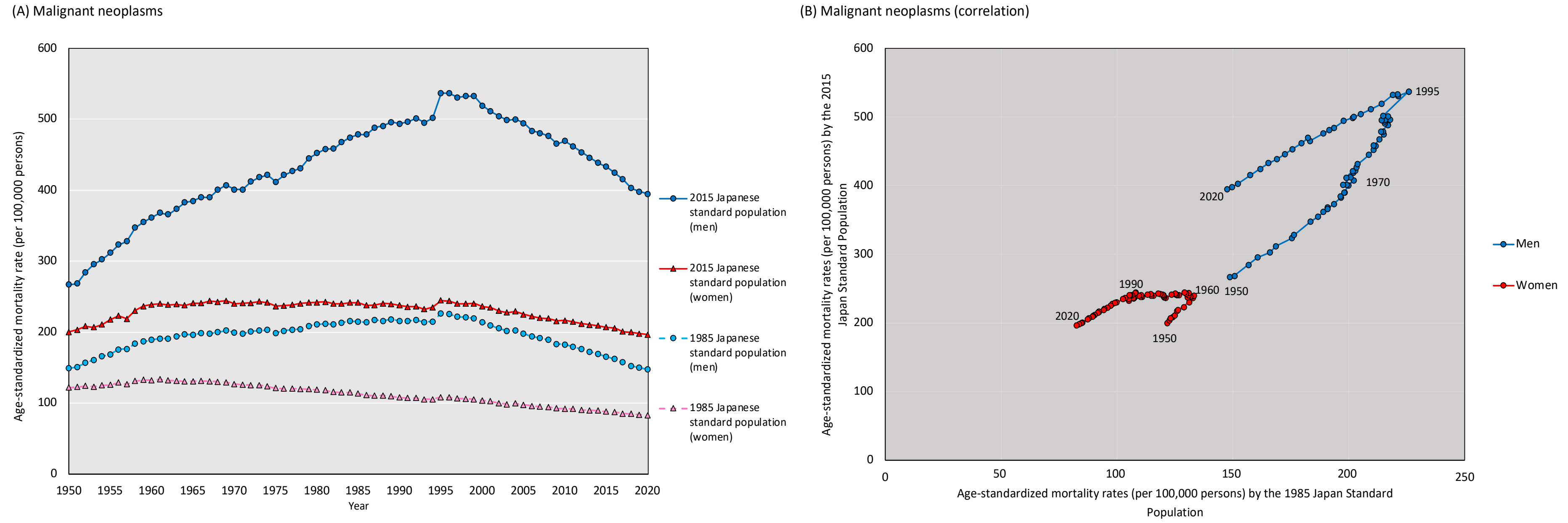

eFigure 2. Trends in **(A)** malignant neoplasms age-standardized mortality rates and **(B)** association of age-standardized mortality rates from malignant neoplasms

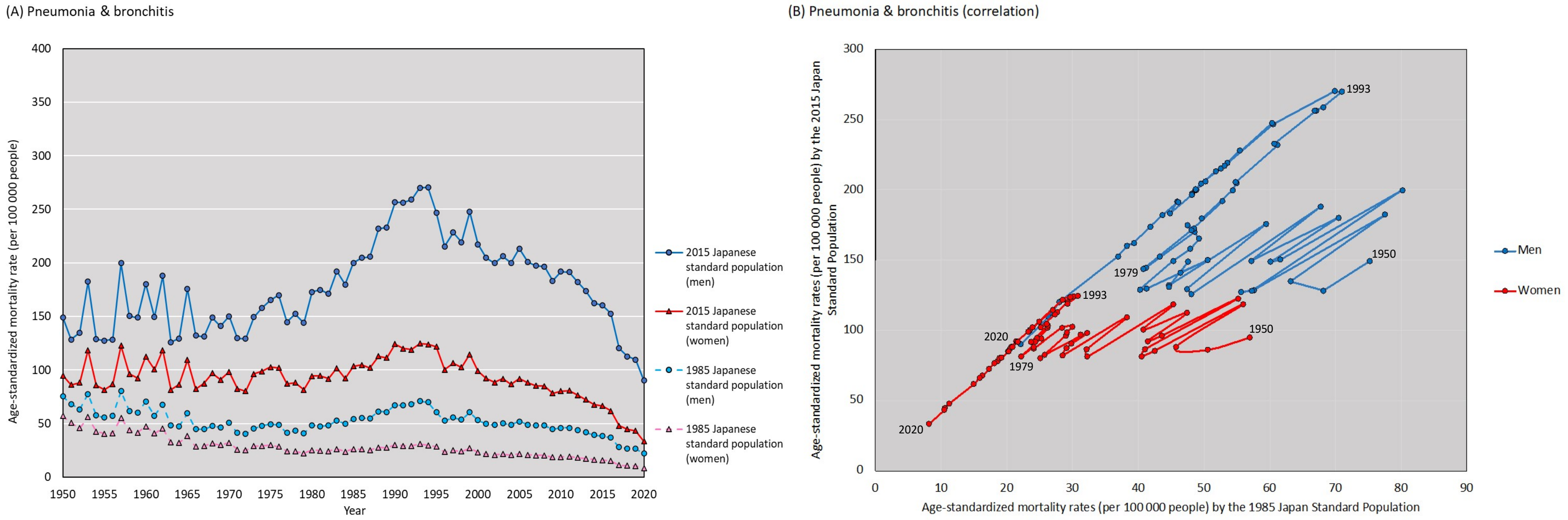

eFigure 3. Trends in **(A)** pneumonia & bronchitis age-standardized mortality rates and **(B)** association of age-standardized mortality rates from pneumonia & bronchitis
